# An Analysis of PubMed Abstracts From 1946 to 2021 to Identify Organizational Affiliations in Epidemiological Criminology: Descriptive Study

**DOI:** 10.2196/42891

**Published:** 2022-12-05

**Authors:** George Karystianis, Wilson Lukmanjaya, Paul Simpson, Peter Schofield, Natasha Ginnivan, Goran Nenadic, Marina van Leeuwen, Iain Buchan, Tony Butler

**Affiliations:** 1 School of Population Health University of New South Wales Sydney, New South Wales Australia; 2 School of Computer Science University of Technology Sydney Sydney Australia; 3 Neuropsychiatry Service Hunter New England Health Newcastle Australia; 4 School of Psychology University of New South Wales Sydney Australia; 5 Department of Computer Science University of Manchester Manchester United Kingdom; 6 University of New South Wales Library University of New South Wales Sydney Australia; 7 Institute of Population Health University of Liverpool Liverpool United Kingdom

**Keywords:** epidemiological criminology, PubMed, offenders, justice health, affiliations, health database, research output, criminology, publication, open research, research promotion, epidemiology research, research database

## Abstract

**Background:**

Epidemiological criminology refers to health issues affecting incarcerated and nonincarcerated offender populations, a group recognized as being challenging to conduct research with. Notwithstanding this, an urgent need exists for new knowledge and interventions to improve health, justice, and social outcomes for this marginalized population.

**Objective:**

To better understand research outputs in the field of epidemiological criminology, we examined the lead author’s affiliation by analyzing peer-reviewed published outputs to determine countries and organizations (eg, universities, governmental and nongovernmental organizations) responsible for peer-reviewed publications.

**Methods:**

We used a semiautomated approach to examine the first-author affiliations of 23,904 PubMed epidemiological studies related to incarcerated and offender populations published in English between 1946 and 2021. We also mapped research outputs to the World Justice Project Rule of Law Index to better understand whether there was a relationship between research outputs and the overall standard of a country’s justice system.

**Results:**

Nordic countries (Sweden, Norway, Finland, and Denmark) had the highest research outputs proportional to their incarcerated population, followed by Australia. University-affiliated first authors comprised 73.3% of published articles, with the Karolinska Institute (Sweden) being the most published, followed by the University of New South Wales (Australia). Government-affiliated first authors were on 8.9% of published outputs, and prison-affiliated groups were on 1%. Countries with the lowest research outputs also had the lowest scores on the Rule of Law Index.

**Conclusions:**

This study provides important information on who is publishing research in the epidemiological criminology field. This has implications for promoting research diversity, independence, funding equity, and partnerships between universities and government departments that control access to incarcerated and offending populations.

## Introduction

Prisoner populations experience poor health, including chronic diseases, exposure to bloodborne viruses, sexually transmissible infections, and mental health problems [1]. Increased all-cause mortality has been described in those exposed to prisons, with the immediate postrelease period a time of heightened vulnerability to suicide and drug overdose [2,3]. The health disparity between prisoners and the general population has been attributed to socioeconomic factors and high-risk health behaviors, including smoking, drinking, and substance use [1,4,5].

Research is necessary to identify the health needs and challenges of prisoners and develop interventions aimed at improving health, welfare, and justice outcomes. The emerging discipline operates at the nexus of the health and criminal justice systems, with a focus on the prevalent health issues that affect offender and incarcerated populations. Epidemiological criminology (or epicriminology) seeks to apply the scientific principles of epidemiology and public health thinking to criminal justice outcomes by framing crime and offending as a public health issue [6]. This involves examining factors such as drug use, mental health, and behavioral conditions to explain and prevent patterns of offending.

Given the increased interest in epicriminology research, it is important to better understand which stakeholders are contributing to this discipline. This may highlight the relative importance that different organizations place on this area and which topics are deemed important to pursue in terms of developing the evidence base. Recognizing who conducts research has implications for impartiality and bias, as it is recognized that those responsible for the development of programs and interventions tend to find more favorable outcomes of such programs than independent evaluators [7]. It may not be in an organization’s best interests to publish negative findings about a program or intervention, but it is important for governments to be accountable to the public they serve; independent university-affiliated researchers may provide such impartiality. Indeed, the independence of research has become a prominent societal issue but generally relates to companies and government agencies that influence research priorities and processes to satisfy investor or political agendas. Perceived independence is an important factor for gaining public trust in research findings [8]. Although independence and conflicts of interest have been extensively discussed in health and medical science literature [8], they remain underexamined in the criminology and justice health fields.

Research productivity is often quantified by summary indices and used to rank countries, institutions, and individuals against each other [9]. This helps inform national and international funding strategies. Universities, perhaps more than other sectors, are highly focused on performance metrics as they impact government, industry, and philanthropic funding and attract students. Research outputs are encouraged to be published in peer-reviewed literature and indexed in large bibliographic databases covering disciplines such as medicine (MEDLINE), sociology (Sociological Abstracts), and psychology (PsychINFO). These, in turn, are accessed by metasearch engines such Scopus, Google Scholar, ProQuest, and LexisNexis, allowing disciplines to be compared between countries, institutions, and individuals. However, niche disciplines such as those focusing on specific populations and emerging fields—as with justice health—tend not to feature in these high-level metrics, thus making it difficult to assess performance.

The advent of big data and the availability of digital data sets makes it possible to conduct large-scale research using those bibliographic databases. PubMed is one such database developed by the National Library of Medicine, which is part of the National Institutes of Health (NIS) and designed to provide access to millions of citations from biomedical journals [10]. For example, there are more than 23,000 articles in the justice health field that report on different epidemiological findings, with more than 13,000 articles published in the last 10 years. However, it is unclear which actors (eg, countries, sectors, and agencies) contribute to this field in terms of peer-reviewed publication outputs.

The aim of this study was to determine the countries and organizations responsible for leading the research in the field of epidemiological criminology. We semiautomatically analyzed the lead author’s affiliation in 23,904 peer-reviewed published outputs from PubMed and mapped them to the World Justice Project Rule of Law Index to better understand how outputs could relate to performance measures of the “functionality” of countries’ justice systems [11].

## Methods

### Research Query

Epidemiological criminology studies are indexed in bibliographical databases related to medicine such as PubMed. Thus, a literature search based on an original query [12] was carried out in PubMed to identify studies relevant to this discipline comprised by 3 parts.

First, we wanted to capture epidemiological studies; thus, we utilized a Medical Subject Headings (MeSH) term (ie, epidemiology) to ensure maximum specificity in the search. Second, since we were focusing on epidemiological studies conducted with offending/incarcerated populations, we used a wide variety of terms that described this marginalized population (eg, “delinquent,” “remandee,” or “offender”) as well as its correctional setting (eg, “prisons,” “correctional facilities,” or “gaols”). This prevented articles that made only passing reference to prison work from entering the data set and resulted in a high-quality corpus for analysis.

Third, to be able to inspect the related affiliations, the search was restricted to English language articles, only as it is the most common language in PubMed.

The full query, which was run on April 20, 2021, was (prison or borstal or jail or jails or gaol or gaols or penitentiary or custody or custodial or (corrective and (service or services)) or ((correctional or detention) AND (centre or centres or center or centers or complex or complexes or facility or facilities)) or (closed AND (setting)) or prisoner or prisoners or incarcerated or criminals or criminal or felon or felons or remandee or remandees or delinquent or delinquents or detainee or detainees or convict or convicts or cellmate or cellmates or offenders or offender or ((young or adolescent) AND (offender or offenders)) or ((delinquent or incarcerated) AND youth) or (juvenile AND (delinquents or delinquent or delinquency or detainee or detainees or offender or offenders)) or ((young) and (people) and (in) and (custody)) or ((justice) and (involved) and (youth)) or ((incarcerated) and (young) AND (people or person or persons)) or ((juvenile or juveniles) and (in) and (custody)) AND english[lang] AND (“epidemiology”[Subheading] or “epidemiology” [MeSH Terms] OR epidemiology [Text Word]).

### Affiliation Processing

We used the PubMed “save” function to download the query results in the “PubMed format.” We automatically processed the files by developing a Python script that identified the first author’s affiliation in each article, as stated under the field “AD,” a designated PubMed heading that indicates affiliation. Usually, the first and last authors belong to the same institute, so we used the first author as a proxy for capturing the institution responsible for carrying out the research.

We automatically added the country associated with the first author’s affiliation to provide a geographical context to the study by searching through a list of countries and determine whether there was a match in the affiliation. Articles with no country in their affiliation were manually inspected by 2 authors (GK and WL), and the country was manually inserted where possible. Articles with countries that no longer exist (eg, Yugoslavia), those belonging to disputed regions (eg, Northern Cyprus), or those with no other information indicated a country were classified as “miscellaneous.”

The affiliations were classified into 5 groups that represent various sectors that conduct research in the epicriminology field:

- The first group comprised universities, including institutes/centers that are part of universities as well as teaching and affiliated hospitals (eg, “The Kirby Institute” is part of the “University of New South Wales” in Australia).
- The second group consisted of prisons, jails, departments of corrective services, and probation and health-related services (administered by departments of corrective services).
- The third group consisted of government (ie, noncorrectional) departments, agencies, and institutes (eg, the “National Institutes of Health” in the United States).
- The fourth group comprised military departments, agencies, and centers including related hospitals and universities (eg, “Second Military Medical School” in China).
- The fifth group consisted of hospitals (public and private), health/medical centers, and clinics that are not affiliated with academia (eg, “Taipei City Hospital” in Taiwan).

The classification was conducted automatically by employing key word search for each group (eg, “university,” “prison”) (Multimedia Appendix 1). Affiliations that could not be mapped to any of these 5 groups were classified as “miscellaneous” (sixth group). An inspection of 50 randomly selected classified affiliations to determine whether they were classified in the wrong group did not return any errors, although it is possible that misclassification could have occurred. If so, these were later rectified after the manual inspection of all classified affiliations (see Data Standardization section).

Affiliations with no identifiable key word were put into the miscellaneous group. All groups were inspected by 2 authors (GK and TB) for misclassification errors. For example, the affiliation *California, Berkeley* refers to the *University of California*, *Berkeley* but did not contain any university-related words. Cases like these were manually assigned the value *University of California, Berkeley* and placed into the appropriate group. This approach was applied to the other 4 groups.

In addition, when authors GK and TB encountered affiliations related to nonprofit organizations (eg, *Médecins Sans Frontieres*) and industry entities including law firms, pharmaceutical corporations, and consultants (eg, *Juniper Associates*), they manually assigned those into 2 new groups that reflected this (“nonprofit organization,” “industry”). Nevertheless, several affiliations (eg, *Center for Criminology*) remained unclassified due to ambiguity or lack of any identifiable information (ie, address, country) and subsequently remained in the “miscellaneous” group. To ensure consistency in this process, we calculated the interannotator agreement as the absolute agreement rate [13] between the 2 annotators (GK and TB) in a random sample of 50 affiliations resulting in 90%, thus suggesting reliable results. Table 1 shows classification examples of first-author affiliations into the 8 groups.

If an article had more than 1 first-author affiliation (marked with the presence of several separators ie, “;,” “/,” “and,” “,”), the affiliations were manually assigned to their respective groups (Multimedia Appendix 2).

**Table 1:** Examples of first-author affiliations that were classified semiautomatically into the 6 initial affiliation groups including those added (ie, industry, nonprofit) after the manual classification.

First-author affiliation	Key word	Affiliation group	Country
School of Psychiatry, University of New South Wales, Sydney, NSW^a^, Australia	University	University	Australia
Indiana women's prison, Indianapolis, Indiana 46214, USA	prison	Prison	United States
Epidemiology unit, Ministry of Health, Gaborone, Botswana	Ministry	Government	Botswana
Mental health department, Israel Defense Forces, Tel Hashomer	Defense	Military	Israel
Rampton hospital, Retford, Notts	hospital	Hospital	United Kingdom
ABT Associated Inc, Cambridge, MA^b^ 02138-1168, USA^c^	N/A^d^	Industry	United States
Médecins Sans Frontières, 7 Bougainvillea Close, Palmerstone, Mutare, Zimbabwe^c^	N/A	Nonprofit organization	Zimbabwe
Centre for Criminology	N/A	Miscellaneous	Unknown

^a^NSW: New South Wales.

^b^MA: Massachusetts.

^c^Originally assigned in the “miscellaneous” group, these were further inspected by authors GK and TB and manually assigned an additional affiliation group (industry, nonprofit).

^d^N/A: not applicable.

### Data Standardization

Each affiliation group was manually inspected by the 2 aforementioned authors (GK and TB) to normalize (when possible) the values of each affiliation and thus enable a suitable presentation of the data for descriptive statistics. Common acronyms were manually expanded (eg, *UNSW* to *University of New South Wales*, *UCL* to *University College London*), synonyms were eliminated (eg, *University of NSW* to *University of New South Wales*), and affiliations that were written in languages other than English (eg, Spanish, Italian) were translated to English (eg, *Universidade Federal do Rio de Janeiro* to *Federal* *University* *of Rio de* *Janeiro*, *Università Cattolica del Sacro Cuore* to *Sacred Heart Catholic University*).

**Figure 1:**
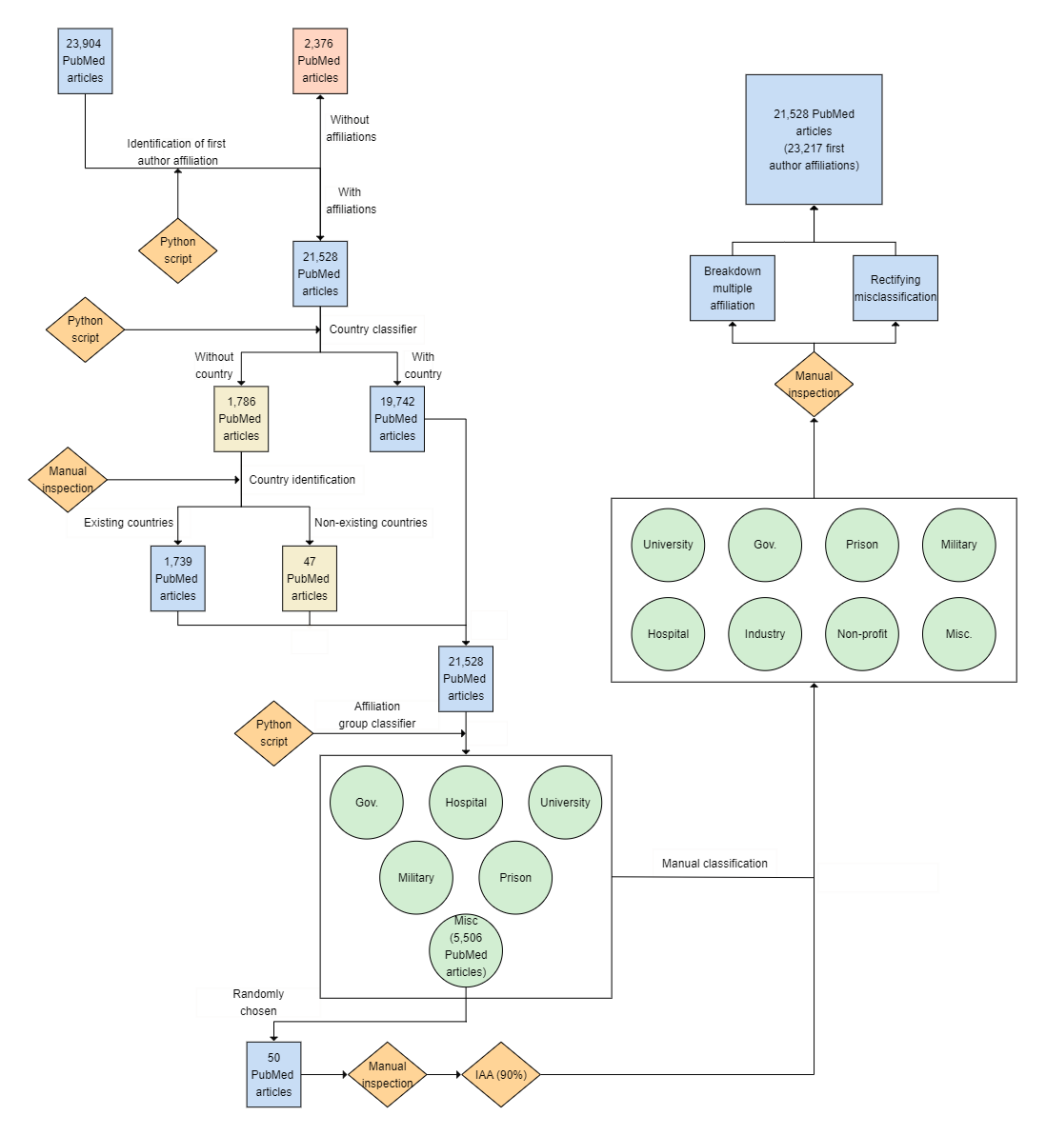
An overview of the semiautomated approach used for the classification and standardization of the first author affiliations from 21,528 PubMed articles. Gov: government; IAA: Inter Annotator Agreement; Misc: miscelleneous.

In addition, some affiliations existed under (or within) specific parent organizations. For example, *National Drug and Alcohol Research Centre, UNSW, Sydney, Australia* was assigned initially into the miscellaneous group, but a manual inspection showed that it is part of the *University of New South Wales*, so its group was changed to university and its value as *University of New South Wales*. Table 2 presents examples of affiliations that were reclassified into other groups following manual inspection. Figure 1 shows an overview of the semiautomated approach that was used to classify and standardize the first-author affiliations.

For reporting purposes, we combined under 1 umbrella term various campuses for big university networks in the United States. For example, affiliations related to the various campuses of *University of California* (ie, San Diego, San Francisco, Berkeley, Davis, Irvine, Los Angeles, Merced, Riverside, Santa Barbara, and Santa Cruz) were all classified as *University of California*.

**Table 2:** First-author affiliations reclassified after manual inspection.

First-author affiliation	Key word	Initial affiliation group	Affiliated institution	New affiliation group	Country
Department of Emergency Medicine, Tri-Service General Hospital, National Defense Medical Center, Taipei, Taiwan	Hospital	Hospital	National Defense Medical Center	Military	Taiwan
National Drug and Alcohol Research Centre, UNSW^a^, Sydney, Australia	N/A^b^	Miscellaneous	University of New South Wales	University	Australia
Mathari Hospital, Ministry of Health, PO^c^ Box 40663, Nairobi, Kenya	Ministry	Government	Mathari Hospital	Hospital	Kenya
Office of Public Health, Louisiana Dept of Health and Hospitals, New Orleans	Hospital	Hospital	Louisiana Department of Health and Hospitals	Government	United States

^a^UNSW: University of New South Wales.

^b^N/A: not applicable.

^c^PO: post office.

## Results

### Query Results

**Figure 2:**
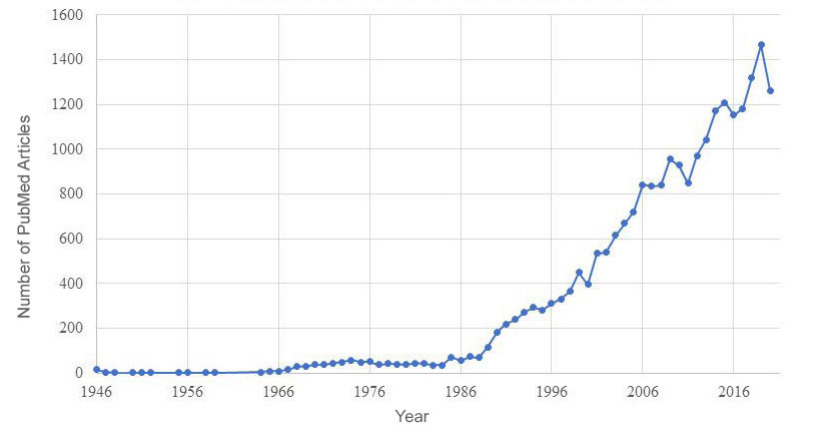
Number of articles related to prisoner health published in PubMed between 1946 and 2020. Since the query was implemented in April 2021, results from that year were not reported.

The query returned 23,904 studies, with the earliest study recorded in 1946. The number of returned studies showed a 95% increase in articles published between 1990 and 2021 (Figure 2).

Almost 1 in 10 articles (n=2376, 9.9%) did not have any author affiliation. Following a manual inspection of 30 randomly chosen articles from the group with no “AD” field, we verified that these articles indeed did not have a first author (or any, for that matter) affiliations, thus reducing our final data set to 21,528 (90.1%) articles (Figure 1). In 1786 (8.2%) articles, the country was manually inserted, and 47 (0.2%) articles had a country status of “miscellaneous.” A total of 5506 (25.5%) affiliations with no identifiable key word were put into the miscellaneous group.

Almost half (n=9188, 42.6%) of the 21,528 articles had first-author affiliations mapped to the United States, followed by United Kingdom (n=2040, 9.4%) and Australia (n=1288, 5.9%) (Table 3). Only 1 country each from South America (Brazil) and Africa (South Africa) appeared in the top 20 publishing countries in epicriminology, whereas Europe had 6 countries in the top 10 (ie, United Kingdom, France, Sweden, Netherlands, Italy, and Germany).

However, to account for the size of the country population, which we assumed to be broadly linked to the size of its prisoner population (Pearson *r*=0.73), and this in turn being a likely driver of research interest reflected by the number of publications, we derived a publication rate based on the average prisoner population size over the period of 2000 to 2020 [14] and calculated a rate per 1000 prisoner population. The rate significantly changed the country ranking in terms of peer-reviewed publication output, with the Nordic entries (ie, Sweden, Finland, Norway, and Denmark) occupying the top 4 spots, while the United States dropped to number 15 (Table 3). When further examining countries that ranked 21 to 30 in terms of peer-reviewed publication outputs and calculating their corresponding publication rate, we found that South Africa, India, Brazil and China were not among the top 20, while Hong Kong (crude rank: 11; publication rate rank: 13.8), Belgium (crude rank: 12; publication rate rank: 13.7), Israel (crude rank: 13; publication rate rank: 9.8), and Greece (crude rank: 14; publication rate rank: 7.9) entered the top 20.

Of the 21,528 articles, 902 (4.2%) had more than 1 first-author affiliation, bringing the total number of affiliations to 23,217. In terms of the affiliation groups responsible for publications across countries, among the 21,528 articles that we examined, first authors affiliated to universities had the highest proportion of peer-reviewed publications (n=15,800, 73.3%) (Table 4). First authors attached to government agencies (n=1928, 8.9%) and hospitals (n=1787, 8.3%) were each responsible for less than 10% of publications, while prison-affiliated first authors were linked to 1% (n=220) of the publications.

A total of 1893 unique universities were identified in our data set. Five countries occupied the top 20 positions with 12 universities based in the United States (Table 5). In terms of crude publication outputs, the *University of California* and *Harvard University* were ranked number 1 and 2, respectively, with the *University of New South Wales* ranking number 3. However, when accounting for the size of the prisoner population in each country, Sweden’s *Karolinska Institute* was ranked the number 1 university in the world in terms of peer-reviewed publication outputs, with the *University of New South Wales* and *University of Melbourne* in second and third place, respectively.

Among the 1928 articles whose first-author affiliation was government related, the *US Centers for Disease Control and Prevention* was the most common government agency, with a publication rate rank of 7 when considering the US prisoner population size (Table 6). The *Norwegian Institute of Public Health* was ranked number 1 (3.6%), followed by the *Justice Health New South Wales (NSW)* (2.9%; Australia) and the *Victorian Institute of Forensic Mental Health* (1.5%; Australia). To more accurately reflect the impact of certain government agencies that have a state focus, we used state prisoner populations rather than national prisoner populations in several instances (see footnote ^e^ in Table 6). For example, the *New York City Department of Health and Mental Hygiene* is likely to serve New York rather than the whole United States.

**Table 3:** Top 20 countries with the highest number of published articles in PubMed (1946-2021) in the epicriminology field along with their respective region, number of articles, prisoner population (average 2000-2020), article rate per 1000 prisoners, and publication rate.

Crude rank	Country	Region	Articles, n (%)	Prisoner population^a^	Article rate per 1000 prisoners^b^	Publicationrate rank
1	United States	North America	9292 (43.2)	2,156,813	4.3	15
2	United Kingdom	Europe	2070 (9.6)	79,564	26	9
3	Australia	Oceania	1289 (6)	30,685	42	5
4	Canada	North America	1077 (5)	38,321	28.1	8
5	France	Europe	493 (2.3)	62,158	7.9	11
6	Sweden	Europe	488 (2.3)	6303	77.4	1
7	Netherlands	Europe	483 (2.2)	14,470	33.4	7
8	Italy	Europe	427 (2)	56,090	7.6	12
9	Germany	Europe	393 (1.8)	68,437	5.7	13
10	China	Asia	367 (1.7)	1,633,561	0.2	20
11	Brazil	South America	346 (1.6)	509,602	0.7	18^c^
12	Spain	Europe	330 (1.5)	61,715	5.3	14
13	India	Asia	267 (1.2)	385,832	0.7	18^c^
14	Switzerland	Europe	250 (1.2)	6257	40	6
15	Finland	Europe	226 (1)	3233	70	2
16	Japan	Asia	215 (1)	65,397	3.3	16
17	Denmark	Europe	195 (0.9)	3742	52.1	4
18	Norway	Europe	192 (0.9)	3289	58.4	3
19	South Africa	Africa	191 (0.9)	164,629	1.2	17
20	New Zealand	Oceania	185 (0.9)	8051	23	10

^a^Average prisoner population 2000 to 2020 (Source: World Prison Brief [14]).

^b^Rate per 1000 prisoners

^c^Equal rank between University of Michigan, University of Maryland, and Emory University.

**Table 4:** Number of PubMed articles (1946-2021) with classified first-author affiliations^a^.

Affiliation group	PubMed articles, n (%)
University	15,800 (73.3)
Government	1928 (8.9)
Hospital	1787 (8.3)
Miscellaneous	953 (4.4)
Nonprofit organization	695 (3.2)
Industry	282 (1.3)
Prison	220 (1)
Military	164 (0.7)

^a^In cases where the first author had more than 1 affiliation listed (eg, a hospital and a university), this was counted as both a hospital and university affiliation unless the hospital was affiliated with the same university, in which case it was counted as 1 affiliation.

**Table 5:** Top 20 universities with the most published articles in PubMed (1946-2021) in the epicriminology field along with their respective country, number of articles, prisoner population (average 2000-2020), and article rate per 1000 prisoners and publication rate.

Crude rank	University	Country	Articles, n (%)	Prisoner population^a^	Article rate per 1000^b^	Publication rate rank
1	University of California	United States	599 (2.8)	2,156,813	0.3	9
2	Harvard University	United States	252 (1.2)	2,156,813	0.1	10
3	University of New South Wales	Australia	246 (1.1)	30,685	8	2
4	Texas University	United States	242 (1.1)	2,156,813	0.1	11
5	Johns Hopkins	United States	239 (1.1)	2,156,813	0.1	12
6	University of Washington	United States	214 (1)	2,156,813	0.1	13
7	Yale University	United States	192 (0.9)	2,156,813	0.1	14
8	Kings College London	United Kingdom	188 (0.9)	79,564	2.4	6
9	Columbia University	United States	184 (0.9)	2,156,813	0.1	15
10	Karolinska Institute	Sweden	184 (0.9)	6303	29.2	1
11	University of North Carolina	United States	179 (0.8)	2,156,813	0.1	16
12	Brown University	United States	159 (0.7)	2,156,813	0.1	17
13	Oxford University	United Kingdom	145 (0.7)	79,564	1.8	7
14	University of British Columbia	Canada	140 (0.7)	38,321	3.7	4
15	University of Toronto	Canada	132 (0.6)	38,321	3.4	5
16	University of Melbourne	Australia	127 (0.6)	30,685	4.1	3
17	Emory University	United Kingdom	119 (0.6)	2,156,813	0.1	18^c^
18	University College London	United States	118 (0.5)	79,564	1.5	8
19	University of Michigan	United States	118 (0.5)	2,156,813	0.1	18^c^
20	University of Maryland	United States	118 (0.5)	2,156,813	0.1	18^c^

^a^Average prisoner population 2000 to 2020 (Source: World Prison Brief [14]).

^b^Rate per 1000 prisoners.

^c^Equal rank between University of Michigan, University of Maryland, and Emory University.

**Table 6:** Top 20 government departments with the most published articles in PubMed (1946-2021) in the justice health field along with their respective country, number of articles, prisoner population (average 2000-2020), article rate per 1000 prisoners, and publication rate.

Crude rank	Government	Country	Articles, n (%)	Prisoner population^a^	Article rate per 1000^b^	Publication rate rank
1	Centers for Disease Control and Prevention	United States	268 (1.2)	2,156,813	0.124	7
2	Justice Health NSW^c^	Australia	35 (0.2)	11,889^d^	2.94	2
3	New York City Department of Health and Mental Hygiene	United States	33 (0.2)	91,000^d^	0.36	5
4	Health Protection Agency	United Kingdom	30 (0.1)	79,564	0.377	4
5	Chinese Centers for Disease Control and Prevention	China	28 (0.1)	1,633,561	0.017	10
6	National Center for Injury Prevention and Control	United States	23 (0.1)	2,156,813	0.011	11
7	National Center for Infectious Diseases	United States	21 (0.1)	2,156,813	0.010	12
8	National Development and Research Institutes	United States	20 (0.1)	2,156,813	0.009	13
9	World Health Organization	World	18 (0.1)	11,500,000^e^	0.002	16
10	Public Health England	United Kingdom	18 (0.1)	79,564	0.226	6
11	National Institute of Health	United States	18 (0.1)	2,156,813	0.008	14
12	National Cancer Institute	United States	17 (0.1)	2,156,813	0.008	14
13	Public Health Service	Netherlands	16 (0.1)	14,470	1.106	4
14	South African Medical Research Council	South Africa	16 (0.1)	164,629	0.097	8
15	Ministry of Social Affairs and Health	Finland	16 (0.1)	3233	0.008	14
16	Ministry of Public Health	Thailand	13 (0.1)	251,695	0.008	14
17	Norwegian Institute of Public Health	Norway	12 (0.1)	3289	3.649	1
18	National Institute on Alcohol Abuse and Alcoholism	United States	11 (0.1)	2,156,813	0.005	15
19	California Department of Health Care Services	United States	11 (0.1)	117,000^d^	0.09	9
20	Victorian Institute of Forensic Mental Health	Australia	10 (0)	6466^d^	1.55	3

^a^Average prisoner population 2000 to 2020 (Source: World Prison Brief [14]).

^b^Rate per 1000 prisoners.

^c^NSW: New South Wales.

^d^Based on available state incarcerated population data.

^e^World prisoner population used (Source: World Prison Brief [14]).

### Publication Rate and the Rule of Law Index

To examine the association between performance measures of justice systems and publication outputs in the justice health arena, we used the 2021 World Justice Project Rule of Law Index [11]. This is a composite index of 8 factors that describe the rule of law through the lens of constraints on government powers, absence of corruption, open government, fundamental rights, order and security, regulatory enforcement, civil justice, and criminal justice [11].

The Index draws on over 400 variables based on country-wide polling and surveys of in-country experts in law and public health, with scores ranging from 0 to 1 (1 being the strongest adherence to the rule of law). Factor 8 of the index focuses on criminal justice and ranks countries based on measures of the effectiveness of criminal justice systems, including whether the “criminal justice system is effective in reducing criminal behavior” and “correctional institutions are secure, respect prisoners’ rights, and are effective in preventing recidivism” [11]. We identified a very high negative correlation (–0.82) between Factor 8 (criminal justice) and the publication rate rank, indicating that countries that ranked the highest in terms of publication rate (eg, Norway, Finland) were also placed higher in terms of the Rule of Law Index (Factor 8) (Denmark: –0.9, Finland: –0.88, Norway: –0.9, Sweden: –0.86).

The bottom 10 ranked countries in the Rule of Law Index (Afghanistan, Cambodia, Democratic Republic of Congo, Egypt, Haiti, Mauritania, Nicaragua, Pakistan, Venezuela, and Cameroon) had a total of 123 publications between 1946 and 2021.

## Discussion

### Principal Findings

The aim of this study was to explore agencies, academic institutions, and industry groups responsible for peer-reviewed, published research outputs in the epicriminology area by analyzing first-author affiliations of PubMed epidemiological studies involving offending and incarcerated populations between 1946 and 2021. We obtained and processed the first-author affiliations of 23,904 PubMed articles using a semiautomated approach to determine which countries produced the most peer-reviewed publications.

Overall, the United States had the highest crude number of published articles in the period between 1946 and 2021, with most from the *University of California* and *Harvard University.* This is consistent with the SCImago Journal and Country rankings, in which the United States leads in terms of citable documents across most subject areas [15]. This is most likely due to the United States having many well-funded universities (second highest number of universities in the world after India [16]) and strong university-industry partnerships (eg, according to SciVal for the period of 2016-2021 in the United States, 4.7% of peer-reviewed publications had an academic-industry collaboration, as opposed to 2.7% for the rest of the world). The United States also has the largest prisoner population in the world, with 25% of the world’s prisoners held in prisons and jails. Therefore, it might be expected to have a greater number of research outputs. However, when the publication rate was calculated based on an estimate of each country’s prisoner population, the United States fell to number 15 overall. Countries with smaller general populations and correspondingly smaller prisoner populations were ranked in the top 10 worldwide in terms of research output. The Nordic countries of Sweden, Finland, Denmark, and Norway occupied the top 4 spots, and Australia ranked fifth. Nordic countries are often regarded as having some of the most progressive approaches to prisoner and offender rehabilitation, with proportionally lower numbers of incarcerated persons and recidivism rates compared to other countries [17-20]. Our findings suggest that conducting research within the prison setting may be a contributing factor in the reduction of recidivism.

We also examined publications in terms of a metric used to rank countries legal systems’ functionality (the Rule of Law Index), which integrates measures of reducing criminal behavior, respecting prisoners’ rights, and recidivism [11]. We found a strong correlation between high scores on the Rule of Law Index and the publication rate rank, suggesting a relationship between publications and country rank in terms of this index. This likely reflects an openness to research and embracing evidence generation by specific countries, which manifests in improved justice outcomes. Countries with lower Rule of Law Index scores had very low corresponding publication rates in our sample, with the lowest 10 (ie, Afghanistan, Cambodia, Cameroon, Democratic Republic of Congo, Egypt, Haiti, Mauritania, Nicaragua, Pakistan, and Venezuela) having a total of only 123 publications between 1946 and 2021. Notably, these nations represent low-income countries with histories of political instability and colonialism that have impeded the translation of economic and social development plans into research activity. Within such a climate, it is unlikely that prisoner health research represents a priority.

We found significant variation in institutions across first-author affiliations, in that 28% (n=6029) of first-author affiliations were not associated with an academic institution. Instead, they were affiliated with government agencies (n=1928, 8.9%) and hospitals/medical centers (n=1787, 8.3%), while 5.3% (n=1141) of the remaining affiliations were linked to nonprofit organizations, the military, and industry. Our findings demonstrate that universities are overwhelmingly responsible (n=15,800, 73.3%) for published peer-reviewed outputs, underscoring their importance and subsequent contribution to the justice health area. This maybe be somewhat surprising, given the Herculean challenges of conducting research in the prison setting [1,3,21]. For example, researchers must navigate multiple ethics committees responsible for providing approvals to conduct research in prison, with approval sometimes taking several years, which could lead to research being abandoned in some cases [21-23].

With universities responsible for undertaking most research in this area and the importance of research independence, a question is raised as to whether government agencies ought to divert funding from their own internal research departments to universities to pursue research on behalf of the public. Identifying the key research groups in a field with poor transparency can potentially enhance dialogue and promote knowledge transfer between universities, government, and prison departments. This can potentially improve health, justice, welfare, and economic outcomes for this highly marginalized population and the community [24].

While first authors from prison-related affiliations represented only 1% (n=220) of our publication data set, this could be due to a preference to conduct in-house research for internal evaluation and consumption. Notwithstanding this, peer review is a marker of research excellence and scientific integrity and an indication that independent expert peers have endorsed the research’s hypotheses, methodology, analytical approach, results, and conclusions and thus ought to be encouraged. However, publications in this area around the effectiveness of applied programs are usually not peer reviewed, mainly because independent researchers may detect negative findings which could reflect poorly on the prison system. However, these are publicly funded agencies; thus, accountability and transparency to the public are imperative. To improve this, program and intervention development should involve universities to minimize the risk of implementing programs with a poor or a nonevidence base and to limit wasting public funds.

### Challenges

The application of a semimanual methodology to classify the first-author affiliation comes with certain challenges. While the first iteration of the classification of affiliations was automated, manually investigating affiliations that remained unclassified (n=5506, 25.5%) and attempting to determine their related group and whether they were part of a larger organization posed a challenge, considering their large number. Several affiliations that were classified as miscellaneous (n=953, 4.4%) had no information (ie, address, type of department, country) that could assist with further identification (eg, *Center for Prisoner and Human Rights*, *Institute of Public Health*), which might have an impact in the order and context of our findings.

This highlights a more generic issue of how problematic the lack of a standardized format in reporting affiliations is. Affiliations are written according to the format of each journal or other publishing authority and might make use of acronyms (eg, *UNSW*, *UCLA*), lack clarity (eg, *HIV/AIDS Asia Regional Program*, *Departments of Emergency Medicine*), refer to only a city or a street address (eg, *Ottawa Ontario*; *2075 Bayview Ave, FG52, Toronto, Ontario, M4N 3M5, Canada, No 25*), neglect to report the affiliation’s country (eg, *National Chung Cheng University*), or describe a certain affiliation in several ways (eg, *University of New South Wales*, *New South Wales University*, *UNSW*, or *University of NSW*).

In addition, some articles (n=1146, 5.3%) had more than 1 first-author affiliation. A specific challenge was to dismantle those, as affiliations can be separated by a semicolon (eg, *University Department of Psychiatry; Royal Edinburgh Hospital, Morningside Park*), a backslash (eg, *Igenomix Valencia/Incliva, Valencia, Spain*), or a connecting preposition (eg, *Naval Medical Center San Diego and University of California San Diego School of Medicine*), among others. To avoid misclassification of these additional affiliations, cases like these were inspected manually. Furthermore, despite focusing only on English results from our PubMed query, some affiliations were written in a different language (ie, Spanish, German, and Indonesian), making it difficult for the authors to manually classify them, especially when acronyms were used (eg, *INSERM*, *CIBERESP*).

These observations indicate that the myriad ways in which affiliations can be reported might cause problems in determining key organizations, thus potentially impacting performance metrics based on affiliation [25]. Such attempts at identifying the necessary organization within an affiliation depend on correct spelling, translation of related affiliations, and appropriate expansion of acronyms, which is what this study attempted to do [26]. Publishing journals should consider adopting a standard or common format (s) for reporting affiliations that at a minimum, reference the lead agency, city, and country.

### Limitations

Our study has several limitations. PubMed articles might not be sufficient to capture an accurate picture for offending and incarcerated populations, as relevant government articles and reports often do not publish in academic journals. Moreover, studies with a more sociological and criminal focus are unlikely to appear in journals covered by PubMed. Thus, our data set likely underestimates the total number of research outputs in this area. In addition, our query may not be broad enough to capture all related articles in this area due to the use of a MeSH term (ie, “epidemiology”). The inclusion of extra MeSH terms such as “clinical trial” and “observational study” could potentially increase the number of articles which could provide potentially a different picture.

The use of first-author affiliations might obscure the true extent of research collaboration and likely underrepresent some groups (eg, prison, nonprofit organizations). Some articles might be the product of a collaboration between different departments and organizations that, while their related research might be conducted by an academic first author, usually contain input from professionals in nonacademic areas that do not necessarily contribute heavily to the publication of academic research. Senior or last author status is often a sought-after spot in a list of authors, and, at this stage, we did not explore this, as we consider the first author to be the person who is (often) responsible for driving the research.

Finally, this study carries the risk of English-language bias because including non-English articles presented resource challenges in terms of prospective costs, time, and expertise in non-English languages. The inclusion of non-English articles would help ensure greater generalizability and reduce bias [27].

### Conclusions

Conducting epidemiological research with offending and incarcerated populations has a well-documented list of challenges. However, for transparency reasons and to identify robust research to improve health and justice outcomes, it is important to understand which types of organizations and agencies are conducting research in this area and quantify how much they contribute to this field. We employed a semiautomated approach to classify the first-author affiliations from 23,904 PubMed epidemiological studies between 1946 and 2021. Nordic countries appear to be generating peer-reviewed output research proportional to their incarcerated population ranking, followed by Australia. Interestingly, more functional legal systems correlated with an increased research output rate. Universities appear to be punching above their weight, with almost three quarters of all published articles in PubMed having first-author affiliations related to a university. *Karolinska Institute* (first rank) and the *University of New South Wales* (second rank) lead the publication rate worldwide, while government departments (n=1928, 8.9%) and prisons (n=220, 1%) were overall in the second and seventh position, respectively. While challenges exist in organizing affiliations into 8 distinct organizational groups, this semimanual meta-analysis provides valuable insights into the epicriminology field that can complement more traditional ranking systems.

## Appendix

Multimedia Appendix 1 Key words used to search and classify the first author affiliations of 21,528 PubMed articles into five groups (university, prison, government, military, hospital).

Multimedia Appendix 2 Examples of first author affiliations with more than one affiliation classified into eight groups.

## Abbreviations

MeSH: Medical Subject Headings
NIH: National Institutes of Health
NSW: New South Wales

## References

[1] Butler T, Schofield P, Detels R, Abdool Karim Q, Baum F, Li L, Leyland AH (2021). Prisoners: a wicked problem for public health. Oxford Textbook of Global Public Health.

[2] Kariminia A, Law MG, Butler TG, Levy MH, Corben SP, Kaldor JM, Grant L (2007). Suicide risk among recently released prisoners in New South Wales, Australia. Med J Aust.

[3] Fazel S, Baillargeon J (2011). The health of prisoners. Lancet.

[4] Pickett K, Wilkinson R (2010). The Spirit Level: Why Equality Is Better for Everyone.

[5] Phelan JC, Link BG, Tehranifar P (2010). Social conditions as fundamental causes of health inequalities: theory, evidence, and policy implications. J Health Soc Behav.

[6] Waltermaurer E, Akers T (2014). Epidemiological Criminology: Theory to Practice.

[7] Lipsey MW (2016). Those confounded moderators in meta-analysis: good, bad, and ugly. AAAPSS.

[8] Gläser J, Ash M, Buenstorf G, Hopf D, Hubenschmid L, Janßen M, Laudel G, Schimank U, Stoll M, Wilholt T, Zechlin L, Lieb K (2021). The independence of research—a review of disciplinary perspectives and outline of interdisciplinary prospects. Minerva.

[9] Almeida RMVR, Borges LFF, Moreira DC, Hermes-Lima M (2020). New metrics for cross-country comparison of scientific impact. Front Res Metr Anal.

[10] Canese K, Weis S (2013). PubMed: the bibliographic database. The NCBI Handbook.

[11] Rule of Law Index. World Justice Project.

[12] Simpson PL, Simpson M, Adily A, Grant L, Butler T (2019). Prison cell spatial density and infectious and communicable diseases: a systematic review. BMJ Open.

[13] Ananiadou S, McNaught J (2006). Text Mining for Biology and Biomedicine.

[14] Average prisoner population 2000 to 2020. World Prison Brief.

[15] Schimago Journal and Country Rank.

[16] Statista.

[17] Johnsen B, Granheim PK, Helgesen J (2011). Exceptional prison conditions and the quality of prison life: Prison size and prison culture in Norwegian closed prisons. European Journal of Criminology.

[18] Lappi-Seppälä T, Tonry M (2011). Crime, criminal justice, and criminology in the Nordic countries. Crime and Justice.

[19] Pratt J, Eriksson A (2014). Contrasts in punishment: an explanation of Anglophone excess and Nordic exceptionalism.

[20] Denny M (2016). Norway's Prison System: Investigating Recidivism and Reintegration. Bridges: A Journal of Student Research.

[21] Lennox C, Leonard S, Senior J, Hendricks C, Rybczynska-Bunt S, Quinn S, Byng R, Shaw J (2022). Conducting randomized controlled trials of complex interventions in prisons: a Sisyphean task?. Front Psychiatry.

[22] Pont J (2008). Ethics in research involving prisoners. Int J Prison Health.

[23] Apa ZL, Bai R, Mukherejee DV, Herzig CTA, Koenigsmann C, Lowy FD, Larson EL (2012). Challenges and strategies for research in prisons. Public Health Nurs.

[24] Ginnivan NA, Chomik R, Hwang YI, Piggott J, Butler T, Withall A (2021). The ageing prisoner population: demographic shifts in Australia and implications for the economic and social costs of health care. IJPH.

[25] Galvez C, Moya-Anegón F (2006). The unification of institutional addresses applying parametrized finite-state graphs (P-FSG). Scientometrics.

[26] Galvez C, Moya-Anegón F (2007). Standardizing formats of corporate source data. Scientometrics.

[27] Jackson JL, Kuriyama A (2019). How often do systematic reviews exclude articles not published in English?. J Gen Intern Med.

