# Supplementary material for: An Analysis of PubMed Abstracts From 1946 to 2021 to Identify Organizational Affiliations in Epidemiological Criminology: Descriptive Study
Source: Interact J Med Res. 2022 Dec 5;11(2):e42891. doi: 10.2196/42891 (PMC9733818; doi:10.2196/42891)
Supplement: Multimedia Appendix 1 [file ijmr_v11i2e42891_app1.docx]

Table S1. Key words used to search and classify the first author affiliations of 21,528 PubMed articles into five groups (university, prison, government, military, hospital).

| Affiliation group | Number of keywords | Related keywords |
| --- | --- | --- |
| University | 2 | universit^a^ , universidad^b^ |
| Prison | 6 | prison, jail, correctional, corrections, penitentiary, corrective |
| Government | 12 | ministry, government, state department, emergency medical service, us department, us agency, cdc, center for disease control, centre for disease control, centers for disease control, centers for diseases control, NHS trust |
| Military | 13 | army, defence force, military, national defence, defense force, armed forces, defense, air force, naval, joint base, defense centers, defence, navy |
| Hospital | 10 | hospital, infirmary, medical center, medical centre, health care, healthcare, health center, health centre, health clinic, medical clinic |

^a^ Used as a string matching for all the variations of the word ‘*university’* in other languages e.g., ‘*universitat*’, ‘*university’*, ‘*universite’*, ‘*universiteit’*

^b^ Used as a string matching for the variations in both Spanish (‘*universidad’*) and Portuguese (‘*universidade*’) of the word ‘*university’*
