# Supplementary material for: An Analysis of PubMed Abstracts From 1946 to 2021 to Identify Organizational Affiliations in Epidemiological Criminology: Descriptive Study
Source: Interact J Med Res. 2022 Dec 5;11(2):e42891. doi: 10.2196/42891 (PMC9733818; doi:10.2196/42891)
Supplement: Multimedia Appendix 2 [file ijmr_v11i2e42891_app2.docx]

Table S2. Examples of first author affiliations with more than one affiliation classified into eight groups.

| First author affiliation | Related affiliations | Keyword | Affiliation group | Country |
| --- | --- | --- | --- | --- |
| University of Birmingham and Lucy Faithfull Foundation, Birmingham, UK | University of Birmingham | university | university | UK |
|  | Lucy Faithfull Foundation^a^ | - | non-profit organization | UK |
| University of Toronto and Juniper Associates, Toronto, Canada | University of  Toronto | university | university | Canada |
|  | Juniper Associates^a^ | - | industry | UK |
| Department of Medical Statistics and Epidemiology, School of Public Health, Sun Yat-Sen University, Guangzhou, Guangdong, PR China, Department of Epidemiology, School of Public health, University of California, Los Angeles, CA, USA | Sun Yat-Sen University | university | university | China |
|  | University of California^a^ | - |  | USA |

^a^ These affiliations were identified during the manual inspection of our dataset, hence the lack of respective keywords.
